# 宣威地区肺癌患者血清和肺组织硒水平研究

**DOI:** 10.3779/j.issn.1009-3419.2011.01.08

**Published:** 2011-01-20

**Authors:** 岚 周, 云超 黄, 竹 王, 联华 叶, 文俊 候, 凯云 杨

**Affiliations:** 1 650118 昆明，昆明医学院第三附属医院，云南省肿瘤医院 The Tird Afliated Hospital of Kunming Medical University, Yunnan Tumor Hospital, Kunming 650118, China; 2 100050 北京，中国预防医学科学院食品与安全所 Institute of Nutrition and Food Safety, Chinese Center for Disease Control and Prevention, Beijing 100050, China; 3 655400 宣威，云南省宣威市人民医院 Xuanwei People's Hospital of Yunnan Province, Xuanwei 655400, China

**Keywords:** 肺肿瘤, 硒, 宣威, Lung neoplasm, Selenium, Xuanwei

## Abstract

**背景与目的:**

宣威是我国肺癌高发区，有研究发现肿瘤的死亡率与硒的地理分布及摄入量呈负相关，肺癌死亡率与血硒水平呈负相关。本研究通过检测宣威肺癌患者的血清硒含量和肺组织硒水平，以初步探讨宣威地区肺癌高发与硒的关系。

**方法:**

取120例宣威女性的血清，其中试验组为60例肺癌患者，对照组为60例非肿瘤非呼吸道疾病人群，测定其血清硒水平。术中取60例肺癌患者的肺癌组织、癌旁肺组织和正常肺组织，其中31例来自于宣威地区，29例来自于非宣威地区，分析癌组织、癌旁组织及正常组织的硒含量。血清和组织硒均采用荧光法进行测定。

**结果:**

宣威地区女性肺癌患者血清硒含量为（55.22±13.34）μg/L，低于对照组（60.33±13.82）μg/L（*P* < 0.05）。宣威地区肺癌组织硒含量为（0.105±0.034）μg/g，低于正常肺组织（0.140±0.048）μg/g（*P* < 0.05）。宣威地区和非宣威地区肺癌组织、癌旁肺组织和正常肺组织间硒水平无统计学差异。腺癌和鳞癌、不同分期的肺癌组织、癌旁肺组织和正常肺组织之间的硒水平均无统计学差异。

**结论:**

宣威地区肺癌的发生与低血硒状态有关，肺组织细胞低硒可能是导致癌变的潜在危险因素。

## Abstract (en)

**Background and objective:**

Xuanwei is an area of the highest incidence and mortality with lung cancer in China. The aim of this study is to determine serum selenium concentrations in lung cancer patients from Xuanwei as well as selenium levels of cancerous tissues, cancer-adjacent pulmonary tissues, and normal pulmonary lung tissues from lung cancer patients, and the relationship between selenium and the high incidence of lung cancer in Xuanwei.

**Methods:**

One hundred and twenty female adults from Xuanwei were enrolled in the study (60 lung cancer patients and 60 with non-tumor and non-respiratory diseases, respectively) and blood samples were collected. Sixty fresh cancerous tissues and their adjacent as well as normal tissues were collected (31 samples from lung cancer patients living in Xuanwei for more than 2 years and 29 from patients in other regions of Yunnan Province outside of Xuanwei, respectively). Serum and tissue selenium concentrations were assayed using a fluorometric method.

**Results:**

Women with lung cancer had a mean serum selenium value (55.22 μg/L±13.34 μg/L) of averagely 8.47%, signifcantly lower than that in subjects with non-tumor and non-respiratory disease controls (60.33 μg/L± 13.82 μg/L)(*P* < 0.05). Selenium concentrations in the tumor tissues (0.105 μg/g±0.034 μg/g) were statistically lower than that of normal ones (0.140 μg/g±0.048 μg/g)(*P* < 0.05) from lung cancer patients in Xuanwei. Statistical differences had not been found between the cases from Xuanwei and non-Xuanwei district, adenocarcinoma and squamous cell carcinoma, among Stage Ⅰ, Stage Ⅱ, stage Ⅲ groups.

**Conclusion:**

Lower serum selenium state was negatively related to the incidence of lung cancer in Xuanwei. It was likely that lower selenium level of lung tissues was potential risk factor to lead to lung cancer.

肺癌是一种严重威胁人类健康和生命的疾病，在全球，肺癌的发病率和死亡率均居恶性肿瘤之首^[1]^。宣威地区位于滇东北，是云南省主要产煤基地，肺癌死亡率为91.11/10万，是我国肺癌高发地区^[2]^。女性发病率高、年轻化、家族聚集性明显是宣威地区肺癌发病的主要特点^[3]^。

硒是人类必需的微量营养素。有研究^[4-6]^发现肿瘤的死亡率与硒的地理分布及摄入量呈负相关，肺癌死亡率与当地人群血硒水平呈负相关^[7, 8]^。据报道宣威肺癌高发区土壤硒的含量低于中国背景值^[9]^，我们推测该地区肺癌高发可能与低硒有关，为此本研究检测了宣威肺癌患者的血清硒含量和肺组织硒水平，以初步探讨宣威地区肺癌高发与硒的关系。

## 1 材料与方法

### 1.1 研究对象

#### 1.1.1 血清

2007年1月-2008年12月间云南省肿瘤医院胸心外科和胸外科就诊的原发性肺癌患者60例，女性，年龄38岁-70岁，平均52岁，经组织和/或细胞病理检查确诊。2007年1月-2008年12月间云南省宣威市人民医院外二科收治的非肿瘤非呼吸道疾病外伤患者60例，女性，年龄35岁-65岁，平均51岁。试验组与对照组均为女性，年龄无统计学差异（*P*=0.444）。两组均在宣威或富源居住2年以上，排除合并糖尿病和肝脏疾病，排除有吸烟史和饮酒史。吸烟史指连续吸烟超过6个月，饮酒史指每周喝酒1次并连续6个月以上。签署知情同意书后，抽取静脉血3 mL置于血清管中，离心取血清1 mL，-80 ℃冰箱保存。

#### 1.1.2 肺组织

2007年1月-2008年12月间云南省肿瘤医院胸心外科和胸外科收治的原发性肺癌患者60例，术中取新鲜肺癌组织、癌旁肺组织（距癌组织边缘2 cm的肺组织）、正常肺组织（距癌组织边缘 > 5 cm的肺组织）。其中宣威地区患者（指在宣威或富源居住2年以上）31例，年龄38岁-62岁，平均48岁；男性10例，女性21例；腺癌21例，鳞癌10例；Ⅰ期15例，Ⅱ期11例，Ⅲ期5例。非宣威地区患者29例，年龄40岁-61岁，平均52岁；男性18例，女性11例；腺癌10例，鳞癌19例；Ⅰ期6例，Ⅱ期18例，Ⅲ期5例。宣威地区和非宣威地区患者年龄无统计学差异，两组均经手术后病理学证实，排除合并其它肿瘤、糖尿病和肝脏疾病。-80 ℃冰箱保存。

### 1.2 主要试剂与仪器

硒标准液、质控猪肉粉、质控小牛血清、DAN试剂、钼酸钠、EDTA、浓盐酸（36%-38%）、氨溶液、环己烷均购自北京化学试剂公司。荧光光谱仪F-7000购自日本Hitachi公司。

### 1.3 配制试剂

0.1%DAN试剂：在暗室内称取DAN 200 mg，加入0.1 mol/L盐酸200 mL，振摇15 min。加入40 mL环己烷，振摇5 min，将此液倒入塞有脱脂棉的分液漏斗中，待分层后滤去环己烷层。纯化后的DAN溶液储于棕色瓶中，加入1 cm厚环己烷覆盖表层。置冰箱内保存。混合酸液：称取钼酸钠（Na_2_MoO_4_·2H_2_O）7.5 g溶于150 mL水中。加入过氯酸（70%-72%）和150 mL去硒硫酸，混匀。EDTA混合液：将A液100 mL、B液10 mL、C液5 mL混合，加水稀释至1, 000 mL。A液（0.2 g/mol EDTA二钠盐）：称取37 g EDTA加热至完全溶解，冷却后稀释至500 mL。B液（10%盐酸羟胺溶液）：称取10 g盐酸羟胺溶于水，稀释至100 mL。C液（0.02%甲酚红指示剂）：称取甲酚红50 mg溶于少量水中，加12.5%氨水1滴，完全溶解后加水稀释至250 mL。

### 1.4 样品消化

常温下解冻血清和组织。称取肺癌组织、癌旁肺组织和正常肺组织各0.5 g。将血清及组织分别放入磨口三角瓶内。加10 mL混合酸液，放置过夜。于沙浴上加热，当激烈反应发生后溶液变为清亮无色并伴有白烟出现，继续加热至溶液呈现黄色，立即取下，冷却后溶液仍转为无色。

### 1.5 生成并测定4, 5-苯并苤硒脑

消化完毕的溶液中加入20 mL EDTA混合液，混匀后溶液呈深粉红色，用氨溶液及浓盐酸调至略成粉红橙色，pH1.5-2.0，于冷水中冷却。以下在暗室内操作：加入DAN试剂3 mL，混匀后置沸水浴中加热5 min。冷却后，加环己烷3.0 mL，振摇4 min。将全部溶液移入分液漏斗，待分层后放掉水层，将环己烷层由分液漏斗上口倾入带盖试管中，于原子荧光光谱仪上用激发光波长376 nm、放射光波长520 nm测定苤硒脑的荧光强度。

### 1.6 绘制硒标准曲线

精确取标准硒溶液（0.05 μg/mL）0 mL、0.2 mL、1.0 mL、2.0 mL及4.0 mL加去离子水至5 mL后，按样品步骤同时进行测定，绘制标准曲线。

### 1.7 质量控制

正式分析样品前，分别取质控小牛血清和猪肉粉，检测标样中的硒含量，以检验荧光光谱仪的精密度和准确度。

### 1.8 计算

每克样品中硒含量（μg）=[标准硒含量（μg）/（标准硒荧光读数-空白荧光读数）]×[（样品荧光读数-空白荧光读数）/样品重量（g）]。

### 1.9 统计学分析

应用SPSS 11.5统计软件进行数据分析。数据采用Mean±SD表示。组间差异比较采用单因素方差分析。以*P* < 0.05为差异有统计学意义。

## 2 结果

### 2.1 血清硒水平

试验组血清硒浓度为（55.22±13.34）μg/ L，对照组为（60.33±13.82）μg/L，两组相比差异有统计学意义（*P*=0.041）。

### 2.2 肺组织硒水平

60例肺癌患者的硒含量为肺癌组织低于癌旁肺组织（*P* < 0.05），癌旁肺组织低于正常肺组织（*P* < 0.05），肺癌组织明显低于正常肺组织（*P* < 0.01）（表 1）。两个地区肺癌组织、癌旁肺组织和正常肺组织间比较，硒水平无统计学差异，宣威地区肺癌组织硒含量低于该地区正常肺组织水平（*P* < 0.05）（表 2）。腺癌和鳞癌、不同分期的肺癌组织、癌旁肺组织和正常肺组织之间的硒水平均无统计学差异。

**1:** 肺癌患者肺组织硒水平 Mean levels of lung tissue Se measurements in lung cancer patients

Lung tissue	Mean±SD	*P*
Carcinoma (*μ*g/g wet weight)	0.107±0.045	0.049^a^, < 0.001^b^
Adjacent (*μ*g/g wet weight)	0.124±0.049	0.018^c^
Normal (*μ*g/g wet weight)	0.145±0.047	
^a^: Difference between carcinoma and adjacent; ^b^: Difference between carcinoma and normal; ^c^: Difference between adjacent and normal.		

**2:** 宣威地区和非宣威地区肺癌患者肺组织硒水平 Mean levels of lung tissue Se measurements in lung cancer patients according to different residence from Xuanwei and non-Xuanwei

Variables	Xuanwei (*n*=31)	Non-Xuanwei (*n*=29)	*P*
Carcinoma (*μ*g/g wet weight)	0.105±0.034^a^	0.108±0.055^b^	0.781
Adjacent (*μ*g/g wet weight)	0.120±0.032	0.128±0.063	0.576
Normal (*μ*g/g wet weight)	0.140±0.048	0.141±0.059	0.908
^a^: Difference between carcinoma and normal from Xuanwei, *P*=0.002; ^b^: Difference between carcinoma and normal from Non-Xuanwei, *P*=0.030.			

## 3 讨论

血清硒含量是反映人体近期硒营养状况的良好指标，受饮食习惯、年龄、性别等多因素的影响。血清硒在85 μg/L以上被认为硒营养状况充足^[10]^。

本研究以宣威女性作为研究对象，排除了性别、吸烟和饮酒等影响因素后，观察到宣威女性肺癌患者及非肿瘤非呼吸道疾病患者平均血清硒含量均低于85 μg/L，宣威女性普遍存在硒营养不足，究其原因，可能与宣威地区地处低硒环境及当地居民的膳食结构有关。宣威肺癌高发区土壤总硒含量为（0.202, 5±0.099, 5）mg/kg，低于中国正常地区土壤硒水平（0.302, 4 mg/kg）^[9]^，环境硒通过食物链影响人体硒营养状况。宣威地区90%以上的人口是农民，膳食内容以植物性食物为主，绝大多数为自种自产的大米、玉米和马铃薯，较少摄入含硒丰富的食物如海产品等。本研究发现肺癌患者平均血清硒含量比对照组低8.47%，提示机体低血硒状态会增加肺癌的发生，低硒是导致宣威肺癌高发的原因或是其结果需进一步探讨。

组织硒含量是反映机体长期硒负荷水平的指标，与血清硒浓度有关。正常成人肺组织硒含量为0.146 μg/g（湿重）^[11]^。本研究对宣威地区和非宣威地区肺癌患者的肺癌组织、癌旁肺组织、正常肺组织硒含量进行了分析，结果发现，宣威地区和非宣威地区肺癌组织硒含量均低于癌旁肺组织和正常肺组织，呈现从低到高的变化趋势，癌组织有过度消耗硒的现象。前期研究^[12]^发现适宜浓度的硒在体外对云南宣威女性肺腺癌细胞系（XWLC-05）具有抑制细胞生长及诱导凋亡的作用，细胞内一定浓度的硒对维持细胞正常功能具有一定保护作用。本研究提示组织细胞低硒可能是导致癌变的潜在危险因素。细胞内适宜浓度的硒通过提高谷胱甘肽过氧化物酶的活性、清除自由基和过氧化物、保护细胞膜功能、稳定DNA结构的稳定性、调节细胞周期促进癌细胞凋亡等机制起到抑癌作用^[13-15]^。

宣威地区肺癌的发生与低血硒状态有关，肺组织细胞低硒可能是导致癌变的潜在危险因素。硒作为营养促癌因素，其作用机制还有待于通过营养调查和营养分析等做进一步研究。

## References

[1] Parkin DM, Bray F, Ferlay J (2005). Global cancer statistics, 2002. CA Cancer J Clin.

[2] Hao JH, Huang YC, Ren HX (2009). Trend analysis of lung cancer mortality in areas with high incidence form 1973 to 2005. Chin General Pract.

[3] He XZ (2000). Coal-fred indoor air pollution and lung cancer genetic susceptibilitythe etiology research of lung cancer in Xuanwei for 22 years. J Pract Oncol.

[4] Shamberger RJ, Frost DV (1969). Possible protective effect of selenium against human cancer. Can Med Assoc J.

[5] Schrauzer GN, White DA, Schneider CJ (1977). Cancer mortality correlation studies--Ⅲ: statistical associations with dietary selenium intakes. Bioinorg Chem.

[6] Li WG, Xie JR, Yu SY (1986). Relationship between the geographical distribution characteristics and selenium levels of primary liver cancer patients in Qidong. Chin J Oncol.

[7] Knekt P, Aromaa A, Maatela J (1990). Serum selenium and subsequent risk of cancer among Finnish men and women. J Natl Cancer Inst.

[8] Knekt P, Marniemi J, Teppo L (1998). Is low selenium status a risk factor for lung cancer?. Am J Epidemiol.

[9] Sun SZ, Cao JX, Li MZ (1995). Studies on the selenium in environment of endemic disease areas-the levels and forms of selenium. J Hygiene Res.

[10] Shor-Posner G, Miguez MJ, Pineda LM (2002). Impact of selenium status on the pathogenesis of mycobacterial disease in HIV-1-infected drug users during the era of highly active antiretroviral therapy. J Acquir Immune Defc Syndr.

[11] Chen Q, Wang NF, Yan SH (1994). Reference values for blood, hair and organ selenium levels in adults of Beijing. Chin J Prev Med.

[12] Zhou L, Huang YC, Lei YJ (2008). Effects of selenium and reduced glutathione on the proliferation and apoptosis of lung cancer cell line XWLC-05. Chin J Lung Cancer.

[13] Fischer JL, Lancia JK, Mathur A (2006). Selenium protection from DNA damage involves a Ref1/p53/Brca1 protein complex. Anticancer Res.

[14] El-Bayoumy K, Das A, Narayanan B (2006). Molecular targets of the chemopreventive agent 1, 4-phenylenebis (methylene)-selenocyanate in human non-small cell lung cancer. Carcinogenesis.

[15] Smith ML, Lancia  JK, Mercer  TI (2004). Selenium compounds regulate p53 by common and distinctive mechanisms. Anticancer Res.

